# Knowledge, attitude, and practices regarding childhood tuberculosis detection and management among healthcare providers in Cambodia: a cross-sectional study

**DOI:** 10.1186/s12879-022-07245-1

**Published:** 2022-03-31

**Authors:** Yom An, Alvin Kuo Jing Teo, Chan Yuda Huot, Sivanna Tieng, Kim Eam Khun, Sok Heng Pheng, Chhenglay Leng, Serongkea Deng, Ngak Song, Sotheara Nop, Daisuke Nonaka, Siyan Yi

**Affiliations:** 1 Sustaining Technical and Analytical Resources (STAR Project), United States Agency for International Development, Phnom Penh, Cambodia; 2 Department of Global Health, Graduate School of Health Sciences, Faculty of Medicine, University of the Ryukyus, Okinawa, Japan; 3 School of Public Health, National Institute of Public Health, Phnom Penh, Cambodia; 4 Saw Swee Hock School of Public Health, National University of Singapore and National University Health System, Singapore, Singapore; 5 National Centre for Tuberculosis and Leprosy Control, Phnom Penh, Cambodia; 6 World Health Organization, Phnom Penh, Cambodia; 7 United States Agency for International Development, Phnom Penh, Cambodia; 8 KHANA Center for Population Health Research, Phnom Penh, Cambodia; 9 Center for Global Health Research, Touro University California, Vallejo, CA USA

**Keywords:** Childhood tuberculosis, Case detection, Knowledge, attitude and practices, Infectious diseases, Southeast Asia

## Abstract

**Background:**

The World Health Organization (WHO) estimated that 29% of global tuberculosis (TB) and almost 47% of childhood TB cases were not reported to national TB programs in 2019. In Cambodia, most childhood TB cases were reported from health facilities supported by the Global Fund to Fight AIDS, Tuberculosis, and Malaria in 2019. This study aimed to compare the healthcare providers' knowledge, attitude, and practices (KAP) on childhood TB case detection in operational districts (ODs) with high and low childhood TB case detection in Cambodia.

**Methods:**

We conducted a cross-sectional study between November and December 2020 among healthcare providers in 10 purposively selected ODs with high childhood TB case detection and 10 ODs with low childhood TB case detection. A total of 110 healthcare providers from referral hospitals (RHs) and 220 from health centers (HCs) were interviewed. We collected information on socio-demographic characteristics, training, and KAP on childhood TB. Pearson's Chi-square or Fisher's exact and Student's *t*-tests were performed to explore the differences in KAP of healthcare providers from ODs with low vs. high childhood TB detection.

**Results:**

Of the 330 respondents, 193 were from ODs with high childhood TB case detection, and 66.67% were from HCs. A significantly higher proportion (46.11%) of respondents from ODs with high childhood TB case detection received training on childhood TB within the past two years than those from low childhood TB case detection ODs (34.31%) (*p* = 0.03). Key knowledge on childhood TB was not significantly different among respondents from ODs with high and low childhood TB case detection. A significantly higher proportion of respondents from ODs with high childhood TB case detection had a good attitude (98.96 vs. 97.08%, *p* = 0.002) and performed good practices (58.55 vs. 45.26%, *p* = 0.02) on contact investigation in the community than those from low childhood TB case detection ODs.

**Conclusions:**

Healthcare providers from ODs with high childhood TB detection had better attitudes and practices towards childhood TB. The attitudes and practices need to be improved among healthcare providers in ODs with low case detection. Further investment in training and experience sharing on childhood TB case detection among healthcare providers is needed to improve childhood TB case detection.

## Background

TB remains a major global health problem. The estimated number of new TB cases in 2019 was 10 million (range 8.9—11.1 million) [1]. The estimated proportion of childhood TB (age < 15 years) incidence was about 12% of the total global TB incidence [1]. Southeast Asia accounted for 44% of the total cases in the same year [2]. In high-income countries, childhood TB constitutes 2% to 7% of all TB cases and is mainly found among immigrant populations [3]. In contrast, childhood TB represents approximately 15% to 20% of all TB cases in low-income countries [3]. A gap in TB case detection is also a big challenge. Based on the World Health Organization (WHO), approximately 29% of the global TB cases of all ages and 47% of TB cases in children under 15 years old were missed due to under-diagnosis and under-reporting [1].

Cambodia is one of the countries on track to reach the 2020 milestone to reduce 20% of TB incidence [2] and has been recently excluded from the global list of TB high-burden countries by WHO from 2021 to 2025 [4]. Despite the transition, the WHO still warrants continued attention, and TB will remain a priority in support. The Global TB Report 2019 estimated that TB incidence in Cambodia in 2018 was 49,000 [2], while only 30,017 cases (61% of the estimated incidence cases) were reported to the national TB program (NTP). Childhood TB was also under-reported in Cambodia. In 2019, 6,247 childhood TB cases were reported to the NTP [5], which equated to 73.5% of the total of 8,500 childhood TB cases estimated by the Global TB Report 2019 [2].

Challenges in detecting childhood TB cases remain noticeable in many countries. Based on the WHO, most countries have included contact investigation as a strategy to detect childhood TB cases in their national guidelines. Nevertheless, some of those activities have not been routinely implemented or scaled up [6]. Some studies have reported that the limited knowledge of healthcare providers remains a barrier for diagnosis, treatment, and prevention of childhood TB [6–8]. A study conducted in Cambodia in 2017 found that only 72.5% of clinicians who worked at TB wards could identify five out of seven main childhood TB screening criteria, and they may not apply their knowledge into the practices [7]. Furthermore, findings from a joint program review in Cambodia in 2019 also highlighted that training on childhood TB was not fully provided and recommended to decentralize childhood TB diagnosis to health center level [9].

In Cambodia, the Global Fund to Fight AIDS, Tuberculosis, and Malaria (Global Fund) is the main funding source to the NTP, allocating the funds to non-governmental organizations (NGOs) for TB program implementation. In 2019, the Global Fund-supported TB programs in 46 operational districts (ODs) (out of 103 ODs), covering 56% of the total population in Cambodia [10]. TB cases notified from the 46 ODs under the Global Fund support were 16,599, equaling 55% of the total 30,017 TB cases notified in 2019 [5]. Of a total of 6,247 childhood TB cases reported in 2019 [5], 4,014 cases (64%) were reported from the 46 ODs under the coverage of the Global Fund. The remaining 34% of childhood TB cases were reported from ODs without the Global Fund's support. Key Global Fund-supported activities contributing to TB case detection included TB screening at high burden communities and pagodas/mosques for key affected populations, fast-track mechanisms to send TB specimens or people with presumptive TB from villages to facilities with GeneXpert MTB/RIF® or X-ray, contact investigation at villages, supervision with onsite training, hospital linkage implementation, orientation and training to private pharmacy staff on presumptive TB cases identification and referrals, and orientation and training on TB case detection and diagnosis.

Healthcare providers' knowledge, attitude, and practices (KAP) are key factors contributing to TB case detection and better management [6–8]. A previous study focused only on clinicians' knowledge on childhood TB and the availability and usage of diagnostic tools for childhood TB [7]. Findings from the joint program review 2019 only highlighted uncompleted roll-out training on childhood TB as a challenge for childhood TB management [9]. In addition, the childhood TB cases detection rate in ODs with the Global Fund's support was almost three times higher than the rate in ODs without the Global Fund's support. Therefore, it is necessary to evaluate healthcare providers' KAP from the settings with high and low childhood TB case detection in Cambodia. This study is the first to compare the healthcare providers' KAP on childhood TB case detection in ODs with the high and low childhood TB case detection in Cambodia. The study's findings will help to inform current TB programs in both sites to improve the interventions, particularly childhood TB case detection.

## Methods

### Study setting

**Fig. 1:**
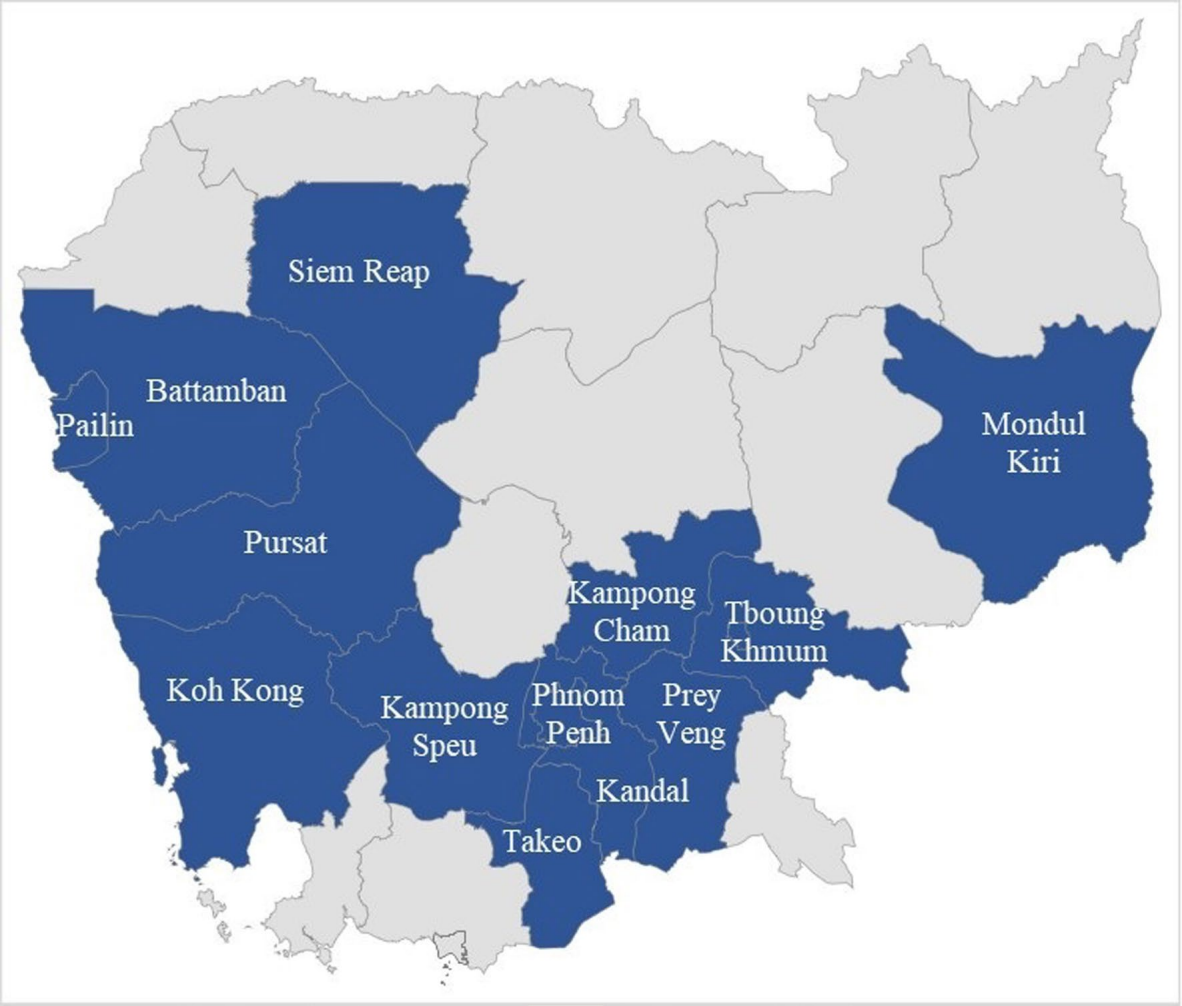
Provinces included in the study

We conducted a quantitative cross-sectional study between November and December 2020. Healthcare providers were recruited from 10 ODs with low and 10 ODs with high childhood TB case detection in 13 provinces (Fig. 1). In each OD, there is one referral hospital covering about 100,000 population and several health centers, each serving about 10,000 population [11]. We purposively selected the 20 ODs to ensure equal representation of urban and rural localities. In the selected study sites, there were a total of 20 referral hospitals and 208 health centers.

### Sample size

At the referral hospitals, the number of healthcare providers varies depending on the level of of the hospital. Complementary package activities 1 (CPA1) refers to referral hospitals with "no grand surgery (without general anesthesia); CPA2 refers to referral hospitals with grand surgery and emergency care services; and CPA3 refers to referral hospitals with grand surgery and various specialized services such as ears, nose, and throat (ENT) and ophthalmology [12]. Six physicians working directly or indirectly on TB in each referral hospital were selected for the interviews. Assuming 10% of the non-response rate, the minimum required sample size was 132. However, if more than six physicians were working directly or indirectly with childhood TB, all of them would be selected for interviews.

In the Minimum Package of Activities (MPA) for Health Center Development 2007, the minimum number of staff required to ensure the proper function of a health center was at least eight people [13]. Healthcare services provided at health centers included outpatient consultation services; maternal, newborn, child, and reproductive health services; infectious diseases services; non-communicable disease services; and health education and health promotion services [13]. In Cambodia, at least two outpatient consultation and communicable disease service staff were directly or indirectly linked to childhood TB services. Therefore, among the 208 health centers in the selected ODs, at least 416 health center staff were eligible (N). The following formula was used to calculated sample size: ${\text{n}} = \, \left[ {{\text{NZ}}^{{2}} {\text{P}}\left( {{1} - {\text{P}}} \right)} \right] \, / \, [{\text{d}}^{{2}} \left( {{\text{N}} - {1}} \right) + {\text{Z}}^{{2}} {\text{P}}\left( {{1} - {\text{P}}} \right)$ [14], where:

n = sample size with finite population correction,

N = population size,

Z = Z statistic for a level of confidence,

P = expected proportion (in study population),

d = precision.

With a 5% margin error (d), 95% confidence interval, and 50% of health center staff correctly identified and referred presumptive childhood TB cases to referral hospitals for TB diagnosis (P), the minimum required sample size was 200. By estimating a 10% non-response rate, the minimum required sample size was 220. With two staff to be selected from one health center, 110 health centers were randomly selected. The selection of health centers was based on the proportion of health centers in each OD.

### Data collection procedures

A questionnaire was initially developed in English and then translated into Khmer. It was pretested with 12 participants who had similar characteristics of final study participants and revised accordingly before being administered by trained data collectors. The interviews were face-to-face using a paper-based questionnaire, and each interview took about 30–40 minutes to complete. Respondents received a compensation gift valued at about one US dollar. The data collection team leader checked the collected data after each interview for completeness and accuracy.

### Variables and measurements

The structured questionnaire was developed based on tools adapted from a previous study [7], a childhood TB treatment guideline training manual developed by the NTP [15], and the current TB-Speed research project implemented by Institut Pasteur du Cambodge through an oral communication with the NTP in February 2020. The tools were tailored to the study settings and objectives through discussions with a research team at the National Center for Tuberculosis and Leprosy Control (CENAT). Sociodemographic characteristics included age, sex, education, role at the health facility (head of the health center or TB staff), departments (infectious diseases, outpatient department or consultation services, medical ward, surgical ward, pediatric ward, or emergency ward), and training on childhood TB. Knowledge on childhood TB included the causes of TB, transmission routes, signs and symptoms, characteristics of lymph nodes that imply TB, and diagnostic criteria for childhood TB. For attitude, we collected participants' perceptions on contact investigation, training on childhood TB, TB diagnostic tools, laboratory services, and human resources for childhood TB. Information on practices included contact investigation performance, presumptive TB referrals for diagnostic work-ups, and TB treatment. Attitude and practices were measured using four levels of the Likert scale [16]. We used four levels of respondents' agreement—"strongly agree," "agree," "disagree," and "strongly disagree")—for each of the seven statements toward childhood TB case detection to measure respondents' attitude. Similarly, four statements about practices toward childhood TB case detection were captured using "always," "often," “sometime,” or “never.”

After data cleaning, recoding was done for some variables. We grouped categories for age, knowledge on TB signs and symptoms, knowledge on groups of children at high risk of developing TB, characteristics of lymph nodes implied TB, and screening criteria for childhood TB to facilitate the subsequent analyses. For attitude, respondents who answered "always" and "often" to the statements were considered to have a positive attitude towards childhood TB case detection. Similarly, good practices were classified when respondents answered “always” and “often” to the positive statements toward childhood TB case detection. Cronbach’s alpha [17] values for internal consistency were 0.856, 0.653, and 0.676 for knowledge, attitude, and practices, respectively.

### Data management and analyses

Collected data were double entered into EpiData 3.1 (The EpiData Association, Odense, Denmark) and then exported into STATA 14.2 (Stata Corp, College Station, Texas). Respondents were categorized into two groups based on whether they were from ODs with high and low childhood TB case detection. Knowledge on childhood TB was categorized as follows: knowing symptoms and signs of childhood TB (< 4 vs. ≥ 4), knowing characteristics of enlarged lymph nodes implying TB (< 3 vs. ≥ 3), and knowing screening criteria for childhood TB (< 4 vs. ≥ 4).

Descriptive analyses were conducted to calculate frequency and proportion for categorical variables and mean and standard deviation (SD) for continuous variables. In bivariate analyses, we used Pearson's Chi-square (or Fisher’s exact test when a cell count was smaller than five) for categorical variables and Student’s *t*-test for continuous variables to explore the differences in characteristics and KAP of healthcare providers from ODs with low vs. high childhood TB detection. A *p* < 0.05 was considered statistically significant.

### Ethical consideration

This study was conducted after obtaining ethical clearances from the National Ethics Committee for Health Research (NECHR) in Cambodia (ref. 234/NECHR) and the Ethics Review Committee of the World Health Organization Western Pacific Regional Office (ID: 2020.8.CAM.3.STB). All methods were carried out in accordance with national guidelines and regulations. The objectives, procedures, risks, and benefits in participating in this study were explained to each participant. All participants provided written informed consent for the study. Data from all participants were treated anonymously, without a name, address, and personal information in the records. Information provided was handled strictly confidential.

## Results

### Sociodemographic characteristics

Sociodemographic characteristics of the study participants are shown in Table 1. In total, we included 193 and 137 respondents from ODs with high and low childhood TB case detection, respectively. About half of the participants were younger than 45 years old, and most were male. A significantly higher proportion of respondents from ODs with high childhood TB case detection received training on childhood TB within the past two years than those with low childhood TB case detection (46.11 vs. 34.31%, *p* = 0.03). Other characteristics did not differ significantly between participants from both sites.

**Table 1:** Sociodemographic characteristics of healthcare providers from ODs with high and low childhood TB case detection

Characteristics	Total (*n* = 330)	ODs with high childhood TB case detection (*n* = 193)	ODs with low childhood TB case detection (*n* = 137)	
	*n* (%)	*n* (%)	*n* (%)	*P*-value
Workplace				0.43
District referral hospital	110 (33.33)	61 (31.61)	49 (35.77)	
Health center	220 (66.67)	132 (68.39)	88 (64.23)	
Age in years				0.58
< 35	107 (32.42)	64 (33.16)	43 (31.39)	
35–44	75 (22.73)	47 (24.35)	28 (20.44)	
45–54	121 (36.67)	65 (33.68)	56 (40.88)	
≥ 55	27 (8.18)	17 (8.81)	10 (7.30)	
Sex, Male	253 (76.67)	142 (73.58)	111 (81.02)	0.12
Working service(s) of respondents from referral hospitals^*^				
Infectious diseases service: TB, HIV, malaria	23 (20.91)	13 (21.31)	10 (20.41)	0.91
Consultation service (OPD)	31 (28.18)	20 (32.79)	11 (22.45)	0.23
Medical service	34 (30.91)	17 (27.87)	17 (34.69)	0.44
Surgical service	13 (11.82)	8 (13.11)	5 (10.20)	0.64
Pediatric service	26 (23.64)	14 (22.95)	12 (24.49)	0.85
Emergency service	24 (21.82)	14 (22.95)	10 (20.41)	0.75
Role of respondents from health centers				0.87
Head of the health center	83 (37.73)	47 (56.63)	36 (43.37)	
OPD staff	30 (13.64)	19 (63.33)	11 (36.67)	
Staff in charge of TB	82 (37.27)	50 (60.98)	32 (39.02)	
Other	25 (11.36)	16 (64.00)	9 (39.00)	
Highest degree of education				0.19
Specialized doctor	8 (2.42)	4 (2.07)	4 (2.92)	
General doctor	101 (30.61)	51 (26.42)	50 (36.50)	
Medical assistant	37 (11.21)	27 (13.99)	10 (7.30)	
Nurse	142 (43.03)	84 (43.52)	58 (42.34)	
Midwife	30 (9.09)	18 (9.33)	12 (2.19)	
Other	12 (3.64)	9 (4.66)	3 (2.19)	
Experience in working with TB or presumptive TB				0.96
< 1 year	113 (43.24)	65 (33.68)	48 (35.04)	
1–5 years	113 (43.24)	65 (33.68)	48 (35.04)	
6–10 years	52 (15.76)	31 (16.06)	21 (15.33)	
> 10 years	52 (15.76)	32 (16.58)	20 (14.60)	
Received training on childhood TB within the past two years	136 (41.21)	89 (46.11)	47 (34.31)	0.03

*OD* operational district, *TB* tuberculosis, *HIV* human immunodeficiency virus, *OPD* outpatient department

*Some of them worked in more than one service

### Childhood TB knowledge

Table 2 summarizes the knowledge of healthcare providers on childhood TB. In general, knowledge of respondents from ODs with high and low childhood TB case detection on causes of TB, routes of TB transmission, groups of children at high risk of developing TB, TB signs and symptoms, characteristics of lymph nodes implying TB, and screening criteria for childhood TB was not significantly different. However, a significantly higher proportion of providers from ODs with low case detection correctly mentioned that weight loss or not gaining weight is a symptom of childhood TB than those with high case detection. Knowledge on the level of fever implying TB (> 38.0 °C) was significantly higher among participants from ODs with high case detection than that among participants from ODs with low case detection.

**Table 2:** Knowledge of healthcare providers on childhood TB

Variables	ODs with high childhood TB case detection (n = 193)	ODs with low childhood TB case detection (n = 137)	
	n (%)	n (%)	P-value
TB is caused by bacteria	171 (88.60)	121 (88.32)	0.94
TB is a transmissible disease	191 (98.96)	137 (100.00)	0.23
TB is spread through expectorated droplet	191 (98.96)	137 (100.00)	0.23
Transmission is reduced after a smear-positive PTB received treatment for two weeks	161 (83.42)	116 (84.67)	0.76
Knowing at least two out of three groups of children at high risk of developing TB below	119 (61.66)	98 (71.53)	0.06
Age less than 1-year-old	48 (24.87)	32 (23.36)	0.75
Living with smear-positive PTB	178 (92.23)	129 (94.16)	0.50
Living with HIV	111 (57.51)	92 (67.15)	0.08
Knew at least four out of eight childhood TB symptoms/signs below	159 (82.38)	118 (86.13)	0.36
Chronic cough	184 (95.34)	126 (91.97)	0.21
Persistent fever	171 (88.60)	118 (86.13)	0.50
Weight loss or not gain weight	175 (90.67)	132 (96.35)	0.046
Night sweats	144 (74.61)	108 (78.83)	0.37
Bone deformity	33 (17.10)	18 (13.14)	0.33
Enlarge lymph nodes	116 (60.10)	95 (69.34)	0.09
Arthralgia	22 (11.40)	17 (12.41)	0.78
Asthenia	83 (43.01)	59 (56.93)	0.99
Knew duration of cough that implies TB (≥ 2 weeks)	156 (80.83)	114 (83.21)	0.58
Knew level of fever that implies TB (> 38.0 °C)	84 (43.52)	40 (29.20)	0.008
Knew at least three out of six characteristics of enlarged lymph nodes implyng TB below	87 (45.31)	62 (45.59)	0.96
Enlarge ≥ 2 cm	139 (72.40)	87 (63.97)	0.10
Painless	86 (44.79)	71 (52.21)	0.19
Asymmetric	105 (54.69)	67 (49.26)	0.33
Firm, matted or discreet	64 (33.33)	40 (29.41)	0.45
Persistent (> 2 weeks)	60 (31.09)	47 (34.31)	0.54
Unresponsive to other treatment (such as antibiotics)	49 (25.52)	33 (24.26)	0.80
Knew at least four out of seven screening criteria for childhood TB below	145 (75.13)	109 (79.56)	0.35
Enlarge lymph nodes	146 (76.04)	110 (80.29)	0.36
Persistent cough	166 (86.46)	119 (86.86)	0.92
Persistent wheezing	22 (11.46)	19 (13.87)	0.51
Weight loss or not gain weight	170 (88.54)	124 (90.51)	0.57
Fever	164 (85.42)	111 (80.02)	0.29
Night sweat	87 (45.08)	62 (45.26)	0.97
Close contact to smear-positive PTB	117 (60.94)	79 (57.66)	0.55
Total knowledge score (total 30), mean (SD)	18.68 (4.74)	18.74 (4.36)	0.90

*TB* tuberculosis, *PTB* pulmonary TB, *TST* tuberculin skin test, *RH* district referral hospital, *HC* health center, *SD* standard deviation

### Attitude toward childhood TB

**Fig. 2:**
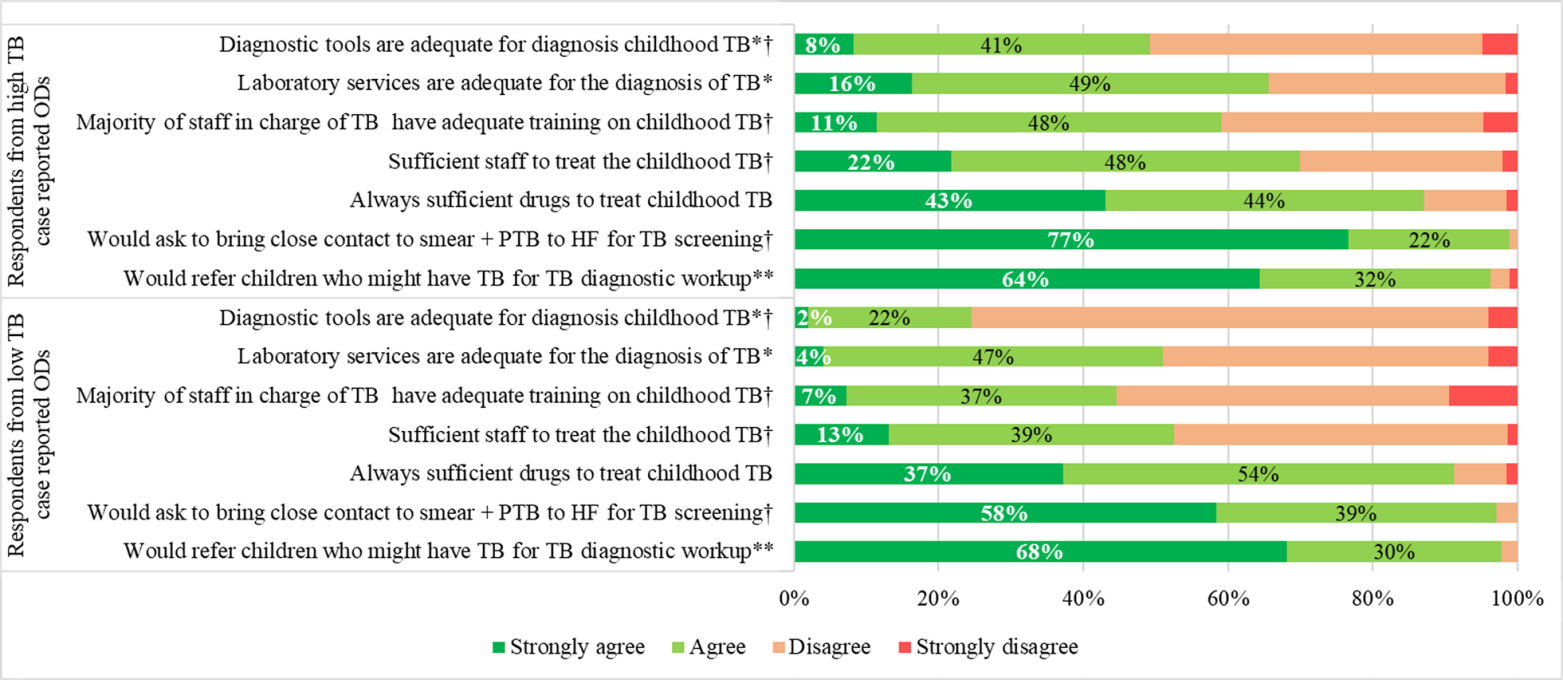
Attitude toward childhood TB among healthcare providers. The horizontal axis of the bar shows the percentages of respondents who strongly agreed, agreed, disagreed, and strongly disagreed with statements used to measure their attitude toward childhood TB. * OD* operational district,* TB* tuberculosis,* PTB* pulmonary TB,* HF* health facilities. ^†^*p* < 0.05. *Respondents from district referral hospitals only. **Respondents from health centers only

The attitude of healthcare providers toward childhood TB are presented in Fig. 2. The proportions of participants who “strongly agreed” or “agreed” that they had received adequate diagnostic tools, training, and staff to treat childhood TB were significantly higher among participants from ODs with high childhood TB case detection than those from ODs with lower childhood TB case detection. Participants from ODs with high childhood TB case detection were also significantly more likely to respond that they would ask people with smear-positive pulmonary TB to bring their close contacts for TB diagnostic work-up. Interestingly, most respondents from health centers in ODs with high and low childhood TB case detection “strongly agreed” or “agreed” to refer children who might have TB for TB diagnosis work-up. Overall, a significantly lower proportion of participants from ODs with low case detection perceived that there were sufficient TB diagnostic tools, training on childhood TB, and staff to treat childhood TB. They were significantly less likely to ask people with smear-positive pulmonary TB to bring close contacts for TB screening.

### Practices of childhood TB

As shown in Table 3, good practices on contact investigation within the community were significantly higher among participants from ODs with high childhood TB case detection than those with low childhood TB case detection (*p* = 0.02). A remarkable higher proportion (76.17%) of participants from ODs with high childhood TB case detection “strongly agreed” or “agreed” to ask index TB patients to bring their close contacts to health facilities for TB screening than those from ODs with low childhood TB case detection (66.42%), although it was not statistically significant (*p* = 0.052). It is noted that a high proportion of respondents from both comparison groups reported good practices on referring children who might have TB for TB diagnostic work-up, 84.46 and 79.56% among participants from ODs with high and low childhood TB case detection, respectively.

**Table 3:** Practices of childhood TB among healthcare providers from ODs with high and low childhood TB case detection

Practice activities	ODs with high childhood TB case detection (*n* = 193)		ODs with low childhood TB case detection (*n* = 137)		
	*n* (%)	*n* (%)	*n* (%)	*n* (%)	*P*-value
Ask index TB patients to bring their close contacts to health facilities for TB screening					
Poor practice	46	23.83	46	33.58	0.05
Good practice	147	76.17	91	66.42	
Perform contact investigation in the community					
Poor practice	80	41.45	75	54.74	0.02
Good practice	113	58.55	62	45.26	
Refer children who might have for TB diagnosis work-up					
Poor practice	30	15.54	28	20.44	0.25
Good practice	163	84.46	109	79.56	
Treat childhood TB					
Poor practice	100	51.81	68	49.64	0.70
Good practice	93	48.19	93	48.19	
Start TB treatment before diagnosis confirmation					
Poor practice*	12	6.22	6	4.38	0.47
Good practice*	181	93.78	131	95.62	

Good practice: Respondents answer “always and often” to the question

Poor practice: Respondents answer “sometime and never” to the question

*TB* tuberculosis, *OD* operational district

*Good practice: Respondents answer “sometime and never” to the question

*Poor practice: Respondents answer “always and often” to the question

## Discussion

This study assessed the KAP of healthcare providers from ODs with high and low childhood TB case detection in Cambodia. The results showed that knowledge on causes of TB, TB transmission routes, symptoms and signs of childhood TB, characteristics of lymph nodes implying TB, and screening criteria for childhood TB were not significantly different among healthcare providers from the OD groups. Most respondents knew that TB is caused by bacteria, similar to findings from a study in Iraq [18, 19] but higher than those in Saudi Arabia [20] and Vietnam [19] and lower than a study in Ethiopia [21]. In our study, knowledge on TB transmission routes was relatively high among healthcare providers from ODs with high childhood TB case detection. This finding is similar to the finding of the study in Iraq [18] and Nigeria [22] but higher than other studies [19–21, 23, 24]. Over 80% of healthcare providers from ODs with high childhood TB case detection knew four or more childhood TB signs and symptoms. Providers’ knowledge of specific TB signs and symptoms is also noticeably high, especially on the four basic TB symptoms (cough, fever, weight loss or not gaining weight, and night sweat).

Knowledge of cough duration implying TB is high in our study, which is similar to a previous study in Cambodia [7], lower than a study in Nigeria [22], and higher than a study in Ethiopia [21]. For fever, although a high proportion of respondents knew that fever is a symptom of TB, a low proportion of them correctly specified the level of fever (> 38.0 °C), implying TB. The proportion is lower than a previous study in Cambodia [7]. Less than half of the total study population knew three or more characteristics of lymph nodes implying TB, which is far lower than findings in a previous study in Cambodia [7]. This difference of knowledge might be due to different target populations selected for the studies. In our study, healthcare providers were selected from both referral hospitals  and health centers, while participants in the previous study were selected only from referral hospitals. Nevertheless, the poor knowledge among health providers indicates the need for training healthcare providers on childhood TB since it has important clinical implications for lymph node TB diagnosis. Knowledge of screening criteria for those who might have TB was relatively high among respondents from both OD groups. However, a low proportion of respondents from both groups knew that a smear-positive pulmonary TB patient's close contact was a screening criterion. Since contact investigation plays a key role in TB case detection [25, 26], healthcare providers must widely understand this.

Healthcare providers’ attitude toward childhood TB case detection is critical. Our results suggest that most respondents had a positive attitude toward childhood TB case detection. Almost all respondents from health centers strongly agreed or agreed to refer children who might have TB for TB diagnosis work-up. However, a significantly higher proportion of providers from ODs with high childhood TB case detection strongly agreed and agreed to bring family members and close contacts of smear-positive pulmonary TB for TB screening. This finding suggests that the attitude toward contact investigation of providers from ODs with low childhood TB case detection needs to be strengthened since contact investigation has been shown as a key intervention contributing to childhood TB case detection [25–31]. A significantly higher proportion of respondents from ODs with high childhood TB case detection strongly agreed that staff in their health facilities received adequate childhood TB training than those from ODs with low childhood TB case detection. Therefore, refresher training on childhood TB should be provided to providers in ODs with high childhood TB case detection. Scientific evidence illustrated that capacity building of frontline health care workers resulted in increasing childhood TB case detection [32–34]. This is also in line with the WHO’s roadmap toward ending TB in children and adolescents to further strengthen healthcare providers’ knowledge on childhood TB [6]. Overall, healthcare providers in this study agreed that there was a lack of human resources for TB screening, indicating the need for more resource allocation in those health facilities.

Healthcare providers from ODs with high childhood TB case detection performed better practices on contact investigation within their health facilities and in the community than those from ODs with low case detection. These better practices could be due to more supports available in facilities under the Global Fund’s coverage and may contribute to higher childhood TB case detection [35, 36]. The NTP should consider strengthening good practices on contact investigation in health facilities and communities in ODs not supported by the Global Fund to improve TB case detection among children [25–31]. Other good practices toward childhood TB case detection, such as referrals of children who might have TB for TB diagnostic work-up, were also higher in ODs with the Global Fund’s supports, although the differences were not statistically significant. Improving these good practices needs to be taken into account to improve childhood TB case detection.

The strengths of this study included the study population and the nature of study design. Respondents selected from ODs with high low childhood TB case detection have similar socio-demographic characteristics such as age, sex, and workplace. In addition, data were collected from healthcare providers at referral hospitals, where childhood TB diagnosis was made and at health centers, the frontline providers who refer presumptive childhood TB for further diagnostic workup at referral hospitals. This allowed us to understand the full picture of healthcare providers’ KAP on childhood TB case detection and management. On the other hand, the nature of the study design allowed us to quickly capture healthcare providers’ KAP that enable the national program to use the findings for the TB program improvement. However, this study has a few limitations. Our primary objective was to identify operational challenges in childhood TB case detection in ODs with reported high and low childhood TB cases. Some respondents from low childhood TB detection ODs received interventions from other organizations during the data collection. So KAP of respondents from ODs with low childhood TB case detection was contaminated by the interventions. However, we found some critical differences between the two groups. Should contamination not occur, we opined that the results would bias away from the null, differences between the two groups would be more apparent. Due to the shortage of staff working in the hospitals, the sample size for respondents from referral hospitals was smaller than the target, limiting the generalizability of the findings. Also, we collected information through face-to-face questionnaire interviews prone to reporting bias. Similarly, we did not measure actual practices by observing staff behaviors in a specific situation or using a simulated client method. Hence, there may have been an overestimation of good practices among providers from both OD groups.

## Conclusions

A similar level of knowledge on childhood TB was identified among healthcare providers from ODs with high and low childhood TB case detection, although a significantly higher proportion of providers with high childhood TB case detection received training on childhood TB within the past two years. However, attitude and practices toward childhood TB case detection were better among healthcare providers from ODs with high childhood TB case detection than those from ODs with low childhood TB case detection. Hence, further investments and efforts should be considered to translate knowledge into action to ensure that children with presumptive TB will be duly screened, detected, and treated in low TB case detection ODs to improve childhood TB detection in Cambodia. This could be done through different mechanisms such as expanding training on childhood TB and providing technical and financial supports to ODs with low childhood TB case detection.

## Data Availability

Data and materials are available based on requests.
