# Correction to: Knowledge, attitude, and practices regarding childhood tuberculosis detection and management among healthcare providers in Cambodia: a cross-sectional study

**DOI:** 10.1186/s12879-022-07359-6

**Published:** 2022-04-12

**Authors:** Yom An, Alvin Kuo Jing Teo, Chan Yuda Huot, Sivanna Tieng, Kim Eam Khun, Sok Heng Pheng, Chhenglay Leng, Serongkea Deng, Ngak Song, Sotheara Nop, Daisuke Nonaka, Siyan Yi

**Affiliations:** 1 Sustaining Technical and Analytical Resources (STAR Project), United States Agency for International Development, Phnom Penh, Cambodia; 2 Department of Global Health, Graduate School of Health Sciences, Faculty of Medicine, University of the Ryukyus, Okinawa, Japan; 3 School of Public Health, National Institute of Public Health, Phnom Penh, Cambodia; 4 Saw Swee Hock School of Public Health, National University of Singapore and National University Health System, Singapore, Singapore; 5 National Centre for Tuberculosis and Leprosy Control, Phnom Penh, Cambodia; 6 World Health Organization, Phnom Penh, Cambodia; 7 United States Agency for International Development, Phnom Penh, Cambodia; 8 KHANA Center for Population Health Research, Phnom Penh, Cambodia; 9 Center for Global Health Research, Touro University California, Vallejo, CA USA

## Correction to: BMC Infectious Diseases (2022) 22:317 https://doi.org/10.1186/s12879-022-07245-1

Following publication of the original article [1], the authors identified an error in the legend of Figure 1.

It currently reads: “Provinces included in the study. OD operational district, TB tuberculosis, PTB pulmonary TB, HF health facilities. ^†^p < 0.05. *Respondents from district referral hospitals only. **Respondents from health centers only”

It should read: “Provinces included in the study”.

The original article [1] has been corrected.
